# Dose-response model of murine typhus (*Rickettsia typhi*): time post inoculation and host age dependency analysis

**DOI:** 10.1186/1471-2334-12-77

**Published:** 2012-03-30

**Authors:** Sushil B Tamrakar, Yin Huang, Sondra S Teske, Charles N Haas

**Affiliations:** 1 Department of Civil, Architectural and Environmental Engineering, Drexel University, Philadelphia, PA 10104, USA

## Abstract

**Background:**

*Rickettsia typhi (R. mooseri) *is the causative agent of murine typhus. It is one of the most widely distributed flea-borne diseases with a relatively mild febrile initial illness with six to 14 days of incubation period. The bacterium is gram negative and an obligate intracellular pathogen. The disease is transmitted to humans and vertebrate host through fleabites or via contact with infected feces. This paper develops dose-response models of different routes of exposure for typhus in rodents.

**Methods:**

Data from published articles were analyzed using parametric dose-response relationship models. Dose-response relationships were fit to data using the method of maximum likelihood estimation (MLE).

**Results:**

Dose-response models quantifying the effects of different ages of rats and time post inoculation in BALB/c mice were analyzed in the study. Both the adult rats (inoculated intradermally) and newborn rats (inoculated subcutaneously) were best fit by exponential models and both distributions could be described by a single dose-response relationship. The BALB/C mice inoculated subcutaneously were best fit by Beta-Poisson models. The time post inoculation analysis showed that there was a definite time and response relationship existed in this case.

**Conclusions:**

Intradermally or subcutaneously inoculated rats (adult and newborn) models suggest that less than 1 plaque-forming unit (PFU) (1.33 to 0.38 in 95% confidence limits) of the pathogen is enough to seroconvert 50% of the exposed population on average. For the BALB/c mouse time post inoculation model, an average dose of 0.28 plaque-forming units (PFU) (0.75 to 0.11 in 95% confidence limits) will seroconvert 50% of the exposed mice.

## Background

Murine typhus, also known as endemic typhus, is one of the most widely distributed flea borne diseases. The causative agent of murine typhus is *Rickettsia typhi*, previously known as *R. mooseri*. It is a relatively mild febrile illness with 6 to 14 days of incubation period [1-3]. It is considered less pathogenic than *R. rickettsii *and *R. prowazekii *(in terms of mortality rate), but *R. typhi *is virulent enough to cause severe infection in the elderly population [3]. The major reservoir of the pathogens is the rat (*Rattus rattus *and *R. norvegicus*) with the rat flea (*Xenopsylla cheopis*) as the main vector. Fleas are infected by transovarian transmission or acquire the contagion while feeding on an infected animal [4]. *R. typhi *is transmitted to the human body or vertebrate host by infected fleabites, or contamination of the broken skin, respiratory tract or conjunctivae of the host with infected feces or tissues during and after flea feeding [2,3].

The flea once acquiring the infection remains infective for life. Interestingly, neither flea nor rat is harmed by the pathogens [2]. Although humans are infected mainly via rat fleas, murine typhus exists endemically in many places where rat and rat fleas are absent [3]. In the United States, the reported cases of murine typhus are focused in south and central Texas, Los Angeles and Orange County, California, where rats and rat fleas are rarely documented. The cat flea/opossum cycle may be one of the possibilities responsible for the disease[5].

The clinical symptoms of infection with *R*. *typhi *in humans are fever, headache, and myalgia. The fever lasts about 12 days in adults with temperature ranges between 102-104F [6]. In severe cases the pathogen can cause meningoencephalitis, interstitial pneumonia and disseminated vascular lesions [7].

Many researchers have reported the response of animals to different doses of *Rickettsia typhi *in order to develop effective therapy and to study the pathology of infected animals. The purpose of this study is to develop dose-response models and to compare the responses in term of age, route of infection and time post inoculation.

## Methods

Since there have been no previously reported dose-response relations, the aim of this study was to extract usable data from the literature and develop dose-response curves. Criteria for data used in our analysis are described as:

• Route of exposure is explicitly stated (such as inhalation, subcutaneous, intradermal, intravenous etc.)

• Methods for dose estimation are described clearly

• The number of subjects for each dose group is stated explicitly

• The number of positive responses for each exposure route is explicitly stated

• The criteria used to define a positive endpoint are stated

• Pathogen is described in detail (source, strain)

• The mode of preparation of pathogenic organisms is described

Aringo-Jaramillo *et al. *(1984) carried out an experiment with *R. typhi *infection in adult and newborn laboratory rats. Nine different doses of *R. typhi *were transdermally and subcutaneously inoculated with seroconversion and death as the responses defined as endpoints [8]. Animals with an indirect fluorescent antibody titer of greater than or equal to 1:40 were considered to be seroconverted [9,10]. However, no animals died.

Crist *et al. *(1984) experimented with *R. typhi *infection in normal and immune mice. Female BALB/c mice were subcutaneously inoculated with various doses of *R. typhi *and seroconversions on different days (after inoculation) were observed [10].

Aringo-Jaramillo *et al. *(1988) conducted experimental inoculation of *R. typhi *in young rats of different age groups. Five different doses were inoculated orally in 3-day, 7 day and 30 day old rats and seroconversion was recorded as the endpoint of response [9].

### Analysis Method

#### Dose-response analysis

Dose response relationships were fit to data using the method of maximum likelihood estimation (MLE) as described in Haas *et al. *(1999). All the data sets are shown in Table 1.

**Table 1:** Data Used

Pathogen/strain	Study/Reference	*Mode of inoculation*	Test animal/Response Organism/Reponses end point	Dose	Number of Test Animals	Positive Responses	Negative Responses
*R. typhi* (Wilmington)	[10]	s.c.	BALB/c mice (sero-conversion on day 9)	0.01(PFU)	10	0	10
				
				0.1	10	0	10
				
				1	10	0	10
				
				10	10	0	10
				
				100	10	0	10
				
				1000	10	0	10
				
				10000	10	10	0

*R. typhi* (Wilmington)	[10]	s.c.	BALB/c mice (sero-conversion on day 12)	0.01(PFU)	10	0	10
				
				0.1	10	0	10
				
				1	10	0	10
				
				10	10	0	10
				
				100	10	7	3
				
				1000	10	8	2
				
				10000	10	10	0

*R. typhi* (Wilmington)	[10]	s.c.	BALB/c mice (sero-conversion on day 15)	0.01(PFU)	10	0	10
				
				0.1	10	0	10
				
				1	10	1	9
				
				10	10	5	5
				
				100	10	9	1
				
				1000	10	9	1
				
				10000	10	10	0

*R. typhi* (Wilmington)	[10]	s.c.	BALB/c mice (sero-conversion on day 21)	0.01(PFU)	10	0	10
				
				0.1	10	0	10
				
				1	10	5	5
				
				10	10	8	2
				
				100	10	9	1
				
				1000	10	10	0
				
				10000	10	10	0

*R. typhi* (Wilmington)	[10]	s.c.	BALB/c mice (sero-conversion on day 28)	0.01(PFU)	10	0	10
				
				0.1	10	3	7
				
				1	10	8	2
				
				10	10	9	1
				
				100	10	10	0
				
				1000	10	10	0
				
				10000	10	10	0

*R. typhi* (Ethiopian)	[8]	i.d.	Adult rat (sero-conversion)	0.0435(PFU)	5	0	5
				
				0.435	5	1	4
				
				4.35	5	5	0
				
				43.5	5	5	0
				
				435	5	5	0
				
				4350	5	5	0
				
				43500	5	5	0

*R. typhi* (Ethiopian)	[8]	i.d.	Newborn rat (sero-conversion)	0.0435(PFU)	8	0	8
				
				0.435	8	2	6
				
				4.35	8	8	0
				
				43.5	8	8	0
				
				435	8	8	0
				
				4350	8	8	0

*R. typhi* (Ethiopian)	[9]	Oral	Young rat 3 day old (sero-conversion)	10(PFU)	3	1	2
				
				100	6	4	2
				
				1000	3	3	0
				
				10000	3	2	1
				
				100000	5	5	0

*R. typhi* (Ethiopian)	[9]	Oral	Young rat 7 day old (sero-conversion)	10(PFU)	3	1	2
				
				100	3	2	1
				
				1000	2	2	0
				
				10000	3	2	1
				
				100000	3	3	0

*R. typhi* (Ethiopian)	[9]	Oral	Young rat 30 day old (sero-conversion)	10(PFU)	3	0	3
				
				100	6	1	5
				
				1000	3	2	1
				
				10000	3	2	1
				
				100000	3	2	1

The statistical programming language, "R" http://www.r-project.org was used for this computation. Two dose response models (exponential and Beta-Poisson) were used [11]. Exponential and Beta-Poisson MLE estimates were made using the BFGS algorithm. Confidence intervals to the best-fit models were determined via bootstrapping with 10,000 bootstrap iterations.

The exponential dose-response model is given in equation (1)

(1) $$P\left(d\right)=1-e^{-kd}$$

where *P (d) *is the probability of response at dose *d *and *k *is the probability that a single organism can survive and initiate infection.

The Beta-Poisson model is given by equation (2)

(2) $$P\left(d\right)=1-{\left[1+\left(\frac{d}{N_{50}}\right)\cdot \left(2^{1\;/)\;\alpha }-1\right)\right]}^{-\alpha }$$

where N_50 _is the median infective dose and α is the slope parameter for the Beta-Poisson model. The equation (2) is derived from the exact Beta-Poisson equation with certain assumptions [11,12].

Goodness of fit for all models was determined by comparing the value of the optimized deviance to the critical χ^2 ^value at degrees of freedom equal to the number of doses minus the number of fitted parameters at a 95% confidence level. Assessment of the statistical significance of improvement of fit that a two parameter model would provide over a single parameter model was made by comparing the reduction in minimized deviance with the critical χ^2 ^value at 1 degree of freedom. Confidence intervals for the best-fit model were estimated via bootstrapping [11].

Pooling analysis was performed for the different animals and bacterial species to ascertain whether the data set had the same underlying distributions. A likelihood ratio test was used to determine if data could be pooled.

### Time post inoculation analysis

In the experiment conducted by Crist and co-investigators with BALB/c mice inoculated subcutaneously with *R. typhi*, the responses were also recorded at different post inoculation times [10]. Huang and Haas (2009) developed dose-response models incorporating time post inoculation as an additional parameter [13]. In the exponential model, the *k *parameter is the probability that a single organism can survive and proliferate in order to initiate a response. It is well known that this is a time-dependent process, and phenomenological responses of animals to bacteria vary not only with the initial dose of microorganisms, but also with the time post inoculation (*TPI*). In the Beta-Poisson model, the *N*_50 _parameter is the dose required to produce a response in 50% of the exposed subjects. Directly related to the growth kinetics of a single organism, the initial dose to elicit response in 50% of the population (*N*_50_) is also expected to vary with the time when the response is observed. To model these effects, Huang *et al. *[14,15] set the parameter *k *and *N*_50 _equal to functions of time.

## Results

### Dose response model for *Rickettsia typhi *(Murine Typhus)

#### Dose-response model of adult rat exposed intradermally to R. typhi, seroconversion as end point of response

**Figure 1:**
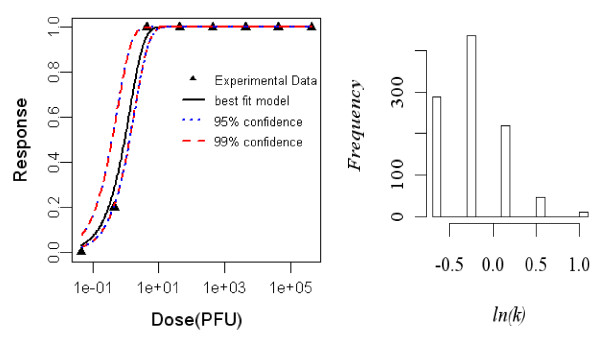
**Dose Response Data and Exponential Model Fits for Seroconversion in Intradermally Exposed Adult rat to *R. typhi *and Bootstrapped Exponential parameters**.

Aringo-Jaramillo *et al. *(1984) studied an experimental infection with *Rickettsia typhi *and antibody response of adult and newborn laboratory rats. The best-fit dose response model for seroconversion in intradermally exposed adult rat was the exponential model. The minimized deviance of the exponential model was 0.88, which was well within the chi-square value at 7 degrees of freedom (i.e. 14.06). The statistics of the two model fits to the animal are summarized in Table 2 and the best-fit model with confidence interval is shown Figure 1.

**Table 2:** Model Fit Comparison for Seroconversion in Intradermally inoculated adult rats

Data set	Number of Doses	Model	Minimized Deviance	Degrees of Freedom	χ_α,n-k_^2^	Parameters	Difference in deviances	*χ*^2 ^Value at 1 degree of freedom
[8]	8	Exponential*	0.88	7	14.06	k = 0.756	0	3.84
			
	8	Beta Poisson	0.88	6	12.59	α = 01.16e8		
								
						N_50 _= 0.91		

*Best fit model

#### Dose-response model of newborn rats exposed subcutaneously to R. typhi, seroconversion as the end point of response

**Figure 2:**
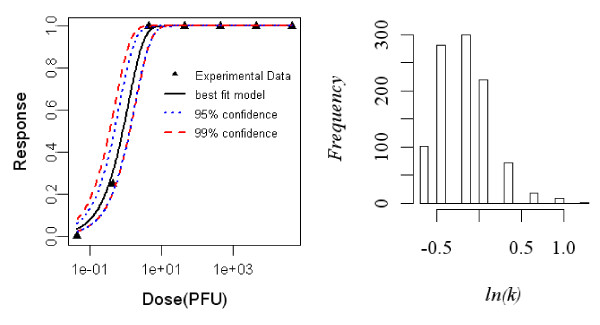
**Dose Response Data and Exponential Model Fits for Seroconversion in Subcutaneously Exposed Newborn rat to *R. typhi *and Bootstrapped Exponential parameters**.

The best-fit dose response model demonstrating seroconversion in subcutaneously exposed newborn rats was the exponential model. The difference in deviances of the Beta-Poisson and the exponential model was zero. Statistics of the two model fits to the animal are summarized in Table 3 and the best-fit model with confidence intervals shown is in Figure 2.

**Table 3:** Model Fit Comparison for Seroconversion in Subcutaneously Exposed Newborn rats

Data set	Number of Doses	Model	Minimized Deviance	Degrees of Freedom	χ_α,n-k_^2^	Parameters	Difference in deviances	*χ*^2 ^Value at 1 degree of freedom
[8]	7	Exponential*	1.12	6	12.59	k = 0.831	0	3.84
			
	7	Beta Poisson	1.12	5	11.07	α = 4.2e7		
								
						N_50 _= 0.83		

*Best fit model

### Dose-response model of rats of different age groups inoculated orally to *R. typhi*, seroconversion as end point of response

Aringo-Jaramillo *et al. *(1988) conducted experimental inoculation of *R. typhi *in young rats of different age groups to study the influence of *R. typhi *in maternal rats has on the offspring. Five different doses were inoculated orally in 3-day, 7-day and 30-day old rats and seroconversion was recorded as the end point of response.

#### Dose-response model of young rat (3-days old) exposed orally to R. typhi, seroconversion as the end point of response

**Figure 3:**
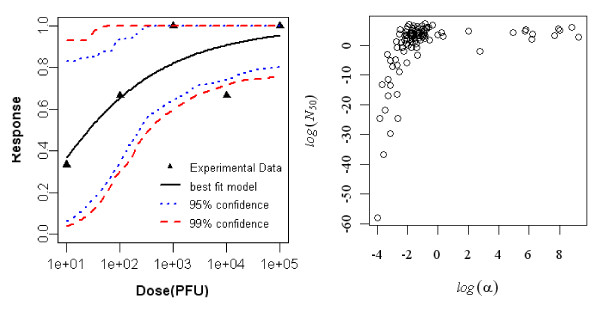
**Dose Response Data and Beta-Poisson Model Fits for Seroconversion in Oral Exposed Young rat (3 day old) to *R. typhi *and Bootstrapped Beta-Poisson Parameters**.

The best fit dose response model for seroconversion in orally exposed young rats (3 day old) was the Beta-Poisson model. The minimized deviance of the exponential model exceeded the chi-square value at 5 degrees of freedom, but that of the Beta-Poisson model was well within the critical chi-square value. Statistics of the two model fits to the animal data are summarized in Table 4 and the best-fit model with confidence intervals is shown in Figure 3.

**Table 4:** Model Fit Comparison for Seroconversion in Oral Exposed Young rat (3 days old)

Data set	Number of Doses	Model	Minimized Deviance	Degrees of Freedom	χ_α,n-k_^2^	Parameters	Difference in deviances	*χ*^2 ^Value at 1 degree of freedom
[9]	5	Exponential	34.63	4	11.07	k = 0.0006	31.60	3.84
			
	5	Beta Poisson*	3.023	3	9.48	α = 0.286		
								
						N_50 _= 25.7		

*Best fit model

#### Dose-response model of young rat (7 day old) exposed orally to R. typhi, seroconversion as the end point of response

**Figure 4:**
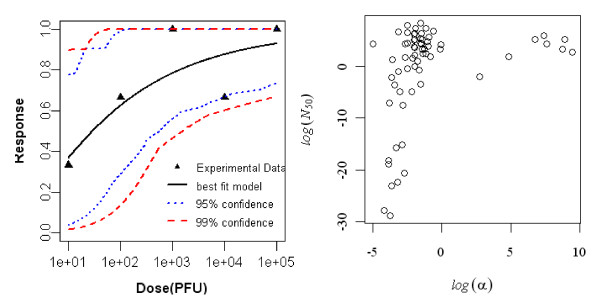
**Dose Response Data and Beta-Poisson Model Fits for Seroconversion in Oral Exposed Young rat (7 day old) to *R. typhi *and Bootstrapped Beta-Poisson parameters**.

The best-fit dose response model for seroconversion in orally exposed young rats (7 days old) was the Beta-Poisson model. Statistics of the two model fits to the data are summarized in Table 5 and the best-fit model with confidence intervals is shown in Figure 4.

**Table 5:** Model Fit Comparison for Seroconversion in Oral Exposed Young rat (7 day old)

Data set	Number of Doses	Model	Minimized Deviance	Degrees of Freedom	χ_α,n-k_^2^	Parameters	Difference in deviances	*χ*^2 ^Value at 1 degree of freedom
[9]	5	Exponential	66.172	4	11.07	k = 0.0149	63.83	3.84
			
	5	Beta Poisson*	2.34	3	9.48	α = 0.241		
								
						N_50 _= 28.92		

*Best fit model

#### Dose-response model of young rat (30 days old) exposed orally to R. typhi, seroconversion as the end point of response

**Figure 5:**
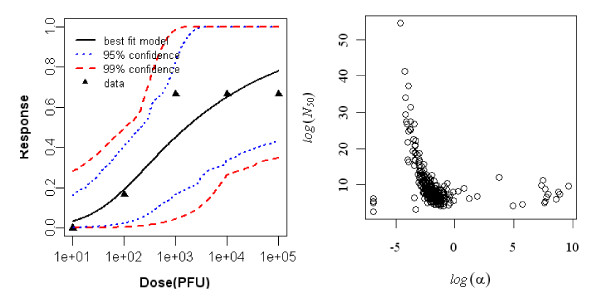
**Dose Response Data and Beta-Poisson Model Fits for Seroconversion in Oral Exposed Young rat (30 day old) to *R. typhi *and Bootstrapped Beta-Poisson parameters**.

The best fit dose response model for seroconversion in orally exposed young rats (30 days old) was the Beta-Poisson model. Statistics of the two model fits to the animal are summarized in Table 6 and the best fit model with confidence intervals is shown in Figure 5.

**Table 6:** Model Fit Comparison for Seroconversion in Oral Exposed Young rat (30 day old)

Data set	Number of Doses	Model	Minimized Deviance	Degrees of Freedom	χ_α,n-k_^2^	Parameters	Difference in deviances	*χ*^2 ^Value at 1 degree of freedom
[9]	5	Exponential	20.55	4	11.07	K = 4.2e-5	19.55	3.84
			
	5	Beta Poisson*	1.0	3	9.48	α = 0.20		
								
						N_50 _= 16.17		

*Best fit model

### Dose-response model with post time inoculation analysis of BALB/c mice exposed subcutaneously to *R. typhi*, seroconversion as the end point of response

**Figure 6:**
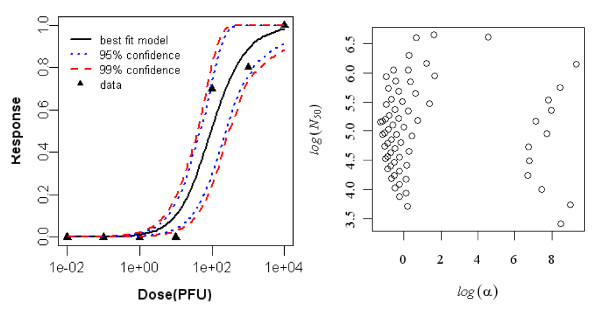
**Dose Response Data and Beta-Poisson Model Fits for Seroconversion (12 days after inoculation) in Subcutaneously Exposed BALB/c mice to *R. typhi *and Bootstrapped Beta-Poisson parameters**.

**Figure 7:**
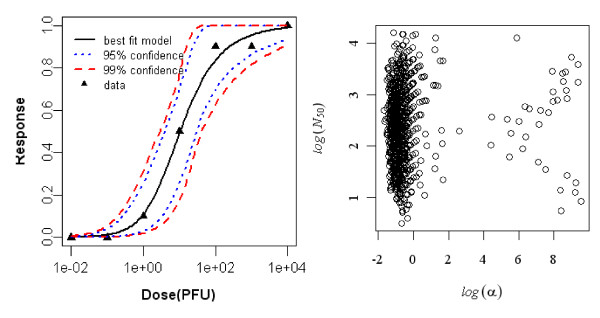
**Dose Response Data and Beta-Poisson Model Fits for Seroconversion (15 days after inoculation) in Subcutaneously Exposed BALB/c mice to *R. typhi *and Bootstrapped Beta-Poisson parameters**.

**Figure 8:**
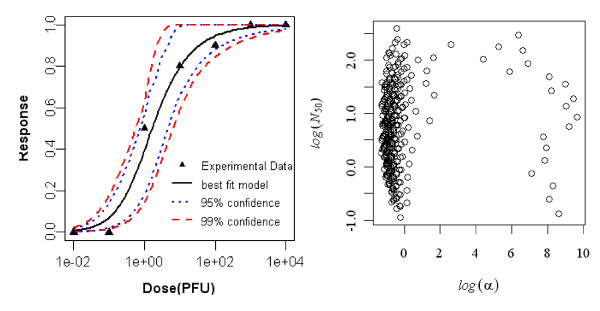
**Dose Response Data and Beta-Poisson Model Fits for Seroconversion (21 days after inoculation) in Subcutaneously Exposed BALB/c mice to *R. typhi *and Bootstrapped Beta-Poisson parameters**.

**Figure 9:**
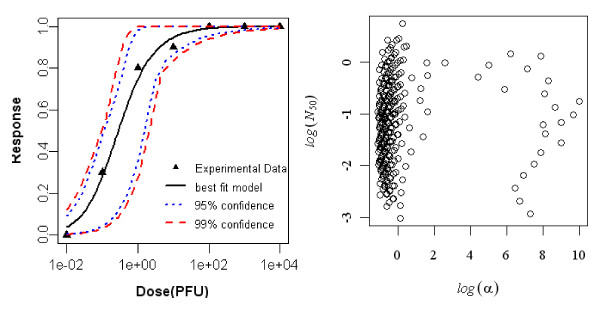
**Dose Response Data and Beta-Poisson Model Fits for Seroconversion (28 days after inoculation) in Subcutaneously Exposed BALB/c mice to *R. typhi *and Bootstrapped Beta-Poisson parameters**.

Crist *et al. *(1984) experimented with *R. typhi (R. mooseri) *infection in normal and immune mice to study the immune mechanism. Seroconversion after 12, 15, 21, and 28 days of inoculation was best fit to the Beta-Poisson models. Seroconversion after 9 days had only two responses and day zero to day 6 had no response at all. In all models, the minimized deviances were well within the chi-square distribution value at five degrees of freedom. Statistics of the two model fits to the animal test data are summarized in Table 7 and the best fit model with confidence intervals is shown in Figures 6, 7, 8 and 9.

**Table 7:** Best fit model for seroconversion on different days after inoculation in subcutaneously exposed BALB/c mice

Data	Number of Doses	Model	Minimized Deviance	Degrees of Freedom	χ_α,n-k_^2^	Parameters
Seroconversion after day 12	7	Beta Poisson*	4.19	5	11.07	α = 0.79
						
						N_50 _= 94.80

Seroconversion after day 15	7	Beta Poisson*	1.40	5	11.07	α = 0.57
						
						N_50 _= 10.46

Seroconversion after day 21	7	Beta Poisson*	2.77	5	11.07	α = 0.60
						
						N_50 _= 1.67

Seroconversion after day 28	7	Beta Poisson*	1.59	5	11.07	α = 0.72
						
						N_50 _= 0.28

*Best fit model

### Time-dose-response model for Murine Typhus in BALB/c mice

Huang *et al. *(Huang, Bartrand et al. 2009; Huang and Haas 2009) proposed a class of time-dose-response models by incorporating the time post inoculation into the classical dose-response models for microbial infection. The parameter *k *in the exponential dose-response model and the parameter *N*_50 _in the Beta-Poisson model were set equal to functions of time, which presumably model the *in vivo *bacterial kinetics for a single microorganism.

The proposed exponential and Beta-Poisson time-dose-response models from these prior studies can be given as:

The exponential and Beta-Poisson model with exponential-reciprocal time dependency-

(3) $$P\left(d,TPI\right)=1-e^{-{e^{{}_{{}^{\left(k_{{}_{0}}/TPI+k_{{}_{1}}\right)}}}}^{{}^{}}d}$$

where the parameter dependency is given by: $g\left(TPI\right)=e^{{}_{{}^{\left(k_{{}_{0}}/TPI+k_{{}_{1}}\right)}}}$

(4) $$P\left(d,TPI\right)=1-{\left[1+\frac{d}{e^{{}_{{}^{\left(j_{{}_{0}}/TPI+j_{{}_{1}}\right)}}}}\times \left(2^{\frac{1}{\alpha }}-1\right)\right]}^{-\alpha }$$

where parameter dependency is given by $g\left(TPI\right)=e^{{}_{{}^{\left(j_{{}_{0}}/TPI+j_{{}_{1}}\right)}}}$The exponential and Beta-Poisson models with power time dependency-

(5) $$P\left(d,TPI\right)=1-e^{-e^{{}_{{}^{\left(k_{{}_{0}}/{\left(TPI\right)}^{k_{2}}+k_{{}_{1}}\right)}}}d}$$

where parameter dependency is given by $g\left(TPI\right)=e^{{}_{{}^{\left[k_{{}_{0}}/{\left(TPI\right)}^{k_{2}}+k_{{}_{1}}\right]}}}$

(6) $$P\left(d,TPI\right)=1-{\left[1+\frac{d}{e^{{}_{{}^{\left(j_{{}_{0}}/{\left(TPI\right)}^{j_{2}}+j_{{}_{1}}\right)}}}}\times \left(2^{\frac{1}{\alpha }}-1\right)\right]}^{-\alpha }$$

where parameter dependency is given by $g\left(TPI\right)=e^{{}_{{}^{\left(j_{{}_{0}}/{\left(TPI\right)}^{j_{2}}+j_{{}_{1}}\right)}}}$

**Figure 10:**
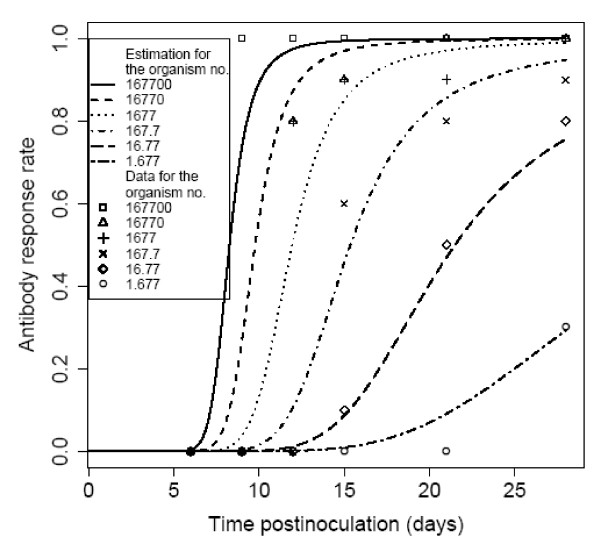
**Time-Dependent Model for Antibody Response of BALB/c mice after subcutaneous inoculation of increasing doses of live *R. mooseri***.

The models with candidate time dependencies [13] were fit to the data of the time-dependent antibody response of mice after subcutaneous inoculation of increasing doses of live *R. typhi *to study the time and dose dependence of the antibody response. The minimum deviances generated by equation (3), (4), (5) and (6) were 109.24, 26.98, 104.66 and 26.47 respectively. The two-parameter modified Beta-Poisson model based on equation (4) provided the best (and statistically acceptable) fit to the data. The best-fit model was then plotted to compare visually with the antibody response in Figure 10. It can be seen that the model is closely aligned with the data.

### Pooling analysis

Pooling analysis was performed to ascertain whether different data sets could be described by a single dose-response relationship. Different combinations of species, strains, routes of infection and hosts were pooled together and a likelihood ratio test was used to determine whether the data could be pooled or not.

**Figure 11:**
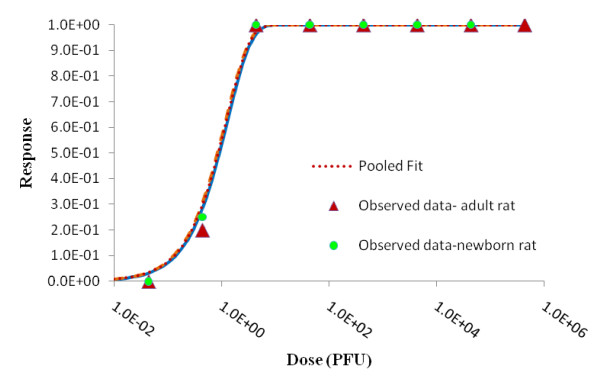
**Best fit model of Adult rat, newborn rat and pooled data**.

The intradermally inoculated adult rats and subcutaneously inoculated newborn rats with *R. typhi *(Ethiopian strain) could be pooled. The difference in deviances between the sum of individual best fits and the pooled model's best fit was zero which was less thanthe *χ*2 _0.05,1 _value (3.841). The summary and statistics of the pooling analysis are shown in Table 8 and the best fit model is shown in Figure 11.

**Table 8:** Adult rat and Newborn rat Pooled Data

Data	Number of Doses	Best fit Model	Minimized Deviance	Degrees of Freedom	χ_α,n-k_^2^	Parameters
*Adult rat exposed intradermally*	8	Exponential	0.88	7	14.06	K = 0.756

*Newborn rat exposed subcutaneously*	7	Exponential	1.12	6	12.59	K = 0.831

*Pooled data*	15	Exponential	2.0	14	23.68	K = 0.801

**Figure 12:**
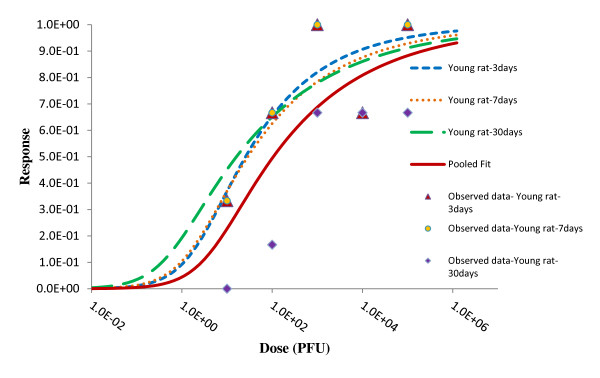
**Best fit models of Young rat (different age group) and pooled data**.

Similarly, young rats of different age groups were orally exposed to graded doses of *R. typhi *orally. All the data could be pooled irrespective of the age of the animals. The value of the difference in deviances between sum of individual best fits and pooled best fit was 0.01 which was less than *χ*2 _0.05,1 _value (3.841). Statistics of the pooled data are summarized in Table 9 and best fit model of the pooled data is shown in Figure 12.

**Table 9:** Young rat of different ages Pooled Data

Data	Number of Doses	Best fit Model	Minimized Deviance	Degrees of Freedom	χ_α,n-k_^2^	Parameters
*Young rat 3-day old*	5	Beta-Poisson	3.023	3	7.81	α = 0.286
						
						N_50 _= 25.7

*Young rat 7-day old*	5	Beta-Poisson	2.34	3	7.81	α = 0.241
						
						N_50 _= 28.92

*Young rat 30-day old *	5	Beta-Poisson	1.0	3	7.81	α = 0.20
						
						N_50 _= 16.17

*Pooled data*	15	Beta-Poisson	13.53	13	22.36	α = 0.213
						
						N_50 _= 106.14

## Discussion

Aringo-Jaramillo *et al. *(1984) studied the antibody response of adult and newborn laboratory rats exposing them intradermally and subcutaneously with *R. typhi*. Both newborn (3 day old) and adult rats were highly susceptible to *R. typhi *inoculated either subcutaneously or intradermally. The ID_50 _for adult rat was 0.91 PFU and for newborn rat was 0.88 PFU. Both routes of infection are considered natural mode of infection analogues to a flea bite[16]. The exponential model provided the best fit in both cases, indicating that the responses were homogenous in the population, and the routes of infection were interchangeable statistically. Moreover, the data could be pooled and as shown in Figure 11, the best fit lines of each of the individual models and the pooled model overlapped one another indicating that responses in rats are independent of the age factor and mode of inoculation (intradermal and subcutaneous). The comparative table as shown in Table 10 also shows there are no significant differences in ID_50_, ID_10 _and ID_01_.

**Table 10:** Comparative values of ID50, ID10 and ID01

Pathogen	Host	Route	ID_01_	ID_10_	ID_50_
*R. mooseri*	Newborn rat	sc	0.012	0.12	0.83
	
	Adult rat	id	0.013	0.14	0.91
	
	Pooled data		0.012	0.13	0.86

sc = subcutaneous

id = intradermal

Similarly, Aringo-Jaramillo *et al. *(1988) studied the influence of maternal *R. typhi *in young rats of different age groups inoculating *R. typhi *orally. All data for every age of rat (3 day-old, 7 day-old and 30 day-old) could be pooled and represented by a best fit Beta-Poisson model. Scattered points at right of the graphs (Figure 6, 7 and 8) are high alpha values and those points tend to fit exponential model while majority 99.9% were at dense cluster. The result indicates that the rats inoculated orally show homogeneity in response and a single dose-response relationship could describe all the age groups. There are no significant differences in ID_50_s of individual age groups but the median infective value is significantly higher than rats inoculated intradermally or subcutaneously. The reason behind the higher ID_50 _may be the route of inoculation. The number of pathogens that reach endothelial cells via the oral route might significantly be less than the initial inoculation.

Crist *et al. *(1984) recorded post time inoculation responses while inoculating *R. typhi (R. mooseri) *to BALB/c mice. In days 0-6, there was no response. There was only one response at the highest dose on day 9. From day 12 to 28, there were systemic responses to corresponding doses. The data of seroconversion of BALB/c mice after days 12, 15, 21 and 28 were best fit to a Beta-Poisson model. The post inoculation effect was analyzed with the data set using a time post inoculation model as described in prior work [13,14]. Figure 10 shows the different dose-response curves for different time (days) after inoculation.

## Conclusion

Human murine typhus caused by *Rickettsia typhi *(*R. mooseri*) is an infectious disease that requires the fewest number of pathogens to initiate disease. The dose-response models developed in this study support this effect. Intradermally or subcutaneously inoculated rats (adult and newborn) dose response models suggest that less than 1 PFU (ranging between 0.38 and 1.33 PFU as estimated within the models' 95% confidence limits) of the pathogen is enough to seroconvert 50% of the exposed population o average. The BALB/c mouse time post inoculation model also indicates that an average dose of 0.28 PFU (0.75 to 0.11 PFU within a 95% confidence interval) will cause seroconversion in of 50% of the exposed population with a mean time to effect of 28 days. The difference in median infectious dose (seroconversion) in adult rat and BALB/c mice is not a significant one and it may be because of the different strains of pathogen used to infect the test subjects. The model suggests that the higher the number of pathogens, the sooner the seroconversion. Pooling analysis of adult and newborn rats shows that there is no significant effect due to different routes of inoculation and can be described by the same dose-response relationship. Similarly, pooling of orally inoculated young rats of different age groups indicates that there is no significant effect of age in serocenversion. However, there is an observed variation in response related to age groups seen in human case studies. According to Al-Awadi *et al.*, the most susceptible age group was the 15-25 years, followed by the 26 to 44 year age group. But there is no definite pattern of variation [17,18]. This is also the first study to incorporate time in a dose-response model for murine typhus. The outcome may improve current understanding of *in vivo *bacterial dynamics, post-exposure decision-making or as a component to assist epidemiological investigations.

## Competing interests

The authors declare that they have no competing interests.

## Authors' contributions

CNH was responsible for conception, acquisition of funding and supervision of the group. SBT made substantial contribution to data collection, study design, analysis and interpretation of data and drafting manuscript. YH contributed to "time post inoculation" section of the manuscript and SST contributed in revising manuscript. All the authors read and approved the final manuscript.

## Pre-publication history

The pre-publication history for this paper can be accessed here:

http://www.biomedcentral.com/1471-2334/12/77/prepub
